# Gait Patterns in Patients with Hereditary Spastic Paraparesis

**DOI:** 10.1371/journal.pone.0164623

**Published:** 2016-10-12

**Authors:** Mariano Serrao, Martina Rinaldi, Alberto Ranavolo, Francesco Lacquaniti, Giovanni Martino, Luca Leonardi, Carmela Conte, Tiwana Varrecchia, Francesco Draicchio, Gianluca Coppola, Carlo Casali, Francesco Pierelli

**Affiliations:** 1 Department of Medico-Surgical Sciences and Biotechnologies, University of Rome Sapienza, Latina, Italy; 2 Rehabilitation Centre, Policlinico Italia, Rome, Italy; 3 Department of Engineering, Roma TRE University, Rome, Italy; 4 Department of Occupational and Environmental Medicine, Epidemiology and Hygiene, INAIL, Monte Porzio Catone, Rome, Italy; 5 Centre of Space Bio-Medicine, University of Rome Tor Vergata, Rome, Italy; 6 Laboratory of Neuromotor Physiology, Istituto Di Ricovero e Cura a Carattere Scientifico Santa Lucia Foundation, Rome, Italy; 7 Department of Systems Medicine, University of Rome Tor Vergata, Rome, Italy; 8 Fondazione Don Gnocchi, Milan, Italy; 9 IRCCS, Neuromed, Pozzilli, Isernia, Italy; 10 G.B. Bietti Foundation-IRCCS, Department of Neurophysiology of Vision and Neurophthalmology, Rome, Italy; Universita degli Studi di Roma 'Foro Italico', ITALY

## Abstract

**Background:**

Spastic gait is a key feature in patients with hereditary spastic paraparesis, but the gait characterization and the relationship between the gait impairment and clinical characteristics have not been investigated.

**Objectives:**

To describe the gait patterns in hereditary spastic paraparesis and to identify subgroups of patients according to specific kinematic features of walking.

**Methods:**

We evaluated fifty patients by computerized gait analysis and compared them to healthy participants. We computed time-distance parameters of walking and the range of angular motion at hip, knee, and ankle joints, and at the trunk and pelvis. Lower limb joint moments and muscle co-activation values were also evaluated.

**Results:**

We identified three distinct subgroups of patients based on the range of motion values. Subgroup one was characterized by reduced hip, knee, and ankle joint range of motion. These patients were the most severely affected from a clinical standpoint, had the highest spasticity, and walked at the slowest speed. Subgroup three was characterized by an increased hip joint range of motion, but knee and ankle joint range of motion values close to control values. These patients were the most mildly affected and had the highest walking speed. Finally, subgroup two showed reduced knee and ankle joint range of motion, and hip range of motion values close to control values. Disease severity and gait speed in subgroup two were between those of subgroups one and three.

**Conclusions:**

We identified three distinctive gait patterns in patients with hereditary spastic paraparesis that correlated robustly with clinical data. Distinguishing specific features in the gait patterns of these patients may help tailor pharmacological and rehabilitative treatments and may help evaluate therapeutic effects over time.

## Introduction

Hereditary spastic paraparesis is a heterogeneous group of inherited neurodegenerative disorders characterized by retrograde degeneration of the corticospinal axonal fibers [1]. Lower limb spasticity, usually more prominent than muscle weakness, is the key clinical feature in patients with hereditary spastic paraparesis [2] and impairs walking ability, autonomy, and quality of life [3,4]. No treatment is known to reduce disease progression, but antispastic drugs and physiotherapy [5–8] may help reduce the functional impairment of gait. Quantifying and typifying the specific gait disorder in hereditary spastic paraparesis is crucial to designing individual pharmacological and rehabilitative treatments. Most descriptions of paraparetic gait are based on qualitative clinical observations [1,2,5], [9–17]. Some studies have quantitatively evaluated gait impairment in hereditary spastic paraparesis patients, revealing several gait abnormalities of reduced step length, increased step width, reduced range of motion (RoM) at the knee joint [18–20], impaired knee torque and stiffness [19,20], and decreased activity of the rectus femoris muscle [19]. Despite the great relevance of such quantitative assessments, they remain generic without reflecting the wide clinical heterogeneity of gait disorders in hereditary spastic paraparesis patients. Spasticity of the lower limb muscles represents the most important clinical sign of hereditary spastic paraparesis, but it affects different patients to different extents [5,18,20]. Individual differences in spasticity should translate into corresponding biomechanical features of gait; specifically, more spastic patients should have more reduced RoMs during walking [18,20]. We hypothesized that the individual kinematic behavior of patients with hereditary spastic paraparesis could be used to identify distinct subgroups of patients, and that these subgroups would exhibit different levels of limb spasticity. Our aims were as follows: i) to perform a comprehensive analysis of kinematics, kinetics and sEMG (surface electromyography) in adult patients with hereditary spastic paraparesis, and ii) to identify specific gait patterns in subgroups of patients categorized according to their kinematic behavior.

## Materials and Methods

### Subjects

We recruited fifty patients with hereditary spastic paraparesis (twenty women and thirty men, mean age 47.70 ± 16.06 years, height 1.64 ± 0.11 m, weight: 75.97 ± 18.51 kg, disease duration 17.65 ± 12.50 years). All patients included in the study were able to walk without assistance or walking aids on a level surface. A defined molecular diagnosis of hereditary spastic paraparesis was applied to thirty patients. Of these, twenty-two patients had spastic paraplegia (SPG) type four (mutations in *SPAST*), two patients had SPG3A (mutations in ATL1), one patient had SPG5 (mutations in *CYP7B1*), two patients had SPG7 (mutations in the *PGN*), and three patients had SPG31 (mutations in *REEP1*). Twenty patients did not have a molecular diagnosis at the time of examination, but all patients unequivocally showed either a recessive (eight patients) or dominant (twelve patients) inheritance pattern. None of the patients showed any involvement of neurological systems other than the pyramidal one (e.g. cerebellar or sensory deficits). All patients were evaluated independently by two experienced neurologists (C.C. and F.P.) who assessed cognitive functions, cranial nerves, muscle tone, muscle strength, joint coordination, tendon reflexes, and sensory function.

The severity of the disease was rated using the Spastic Paraplegia Rating Scale (SPRS). The spasticity of hip and knee joint muscles was scored by the Modified Ashworth scale included in SPRS as a spasticity-related subscale [21]. Table 1 summarizes the clinical features and genotypes of all patients. Twelve out of fifty patients were assuming oral antispastic drugs (baclofen or tizanidine) since 4–6 years, All patients were clinically stable at the time of the study evaluation. Indeed, their clinical assessment (SPRS) did not change over the last six months prior to the study. At the time of the evaluation, all patients were undergoing physical therapy, which included lower limb and stretching exercises, balance, and gait training.

**Table 1:** Patients’ characteristics.

Patients	Gender	Heigth(cm)	Body Wt.(Kg)	Age(yr)	Diagnosis	Onset(yr)	Duration(yr)	SPRS		
								ASHhip	ASHknee	Tot.
p1	M	166	80	67	__AR	30	37	3	3	21
p2	F	158	48	39	__AD	17	22	1	2	16
p3	F	156	66	57	SPG5	36	21	2	2	20
p4	M	178	84	37	SPG4	—	—	0	0	0
p5	F	145	76	50	__AD	30	20	3	3	21
p6	F	146	70	54	__AD	45	9	1	1	6
p7	F	160	62	72	__AD	60	12	2	2	6
p8	F	154	57	66	SPG4	30	36	0	1	6
p9	M	183	75	35	__AD	13	22	1	1	7
p10	M	164	75	56	SPG4	45	11	1	1	12
p11	F	163	58	21	SPG4	3	18	1	1	2
p12	M	162	63	66	SPG4	34	32	3	3	35
p13	F	152	78	40	SPG4	—	—	0	0	0
p14	M	160	57	34	SPG4	1–2	33	3	3	25
p15	M	174	87	47	SPG4	35	12	0	1	7
p16	M	150	62	67	__AD	56	11	1	2	6
p17	M	164	76	67	__AR	45	22	3	3	21
p18	M	170	73	58	SPG4	45	13	1	2	27
p19	M	177	104	24	SPG4	14	10	1	2	11
p20	M	170	88	48	__AR	10	38	1	2	13
p21	M	180	85	25	__AD	13	12	0	0	3
p22	M	162	62	24	__AR	12	12	3	3	21
p23	M	182	109	49	SPG4	37	12	2	2	21
p24	F	170	69	43	SPG4	38	5	0	0	5
p25	F	158	69	72	SPG4	40	32	1	3	31
p26	F	162	58	43	SPG4	5	38	1	2	7
p27	F	142	56	78	SPG4	45	33	2	3	28
p28	M	170	70	49	__AD	24	25	3	3	22
p29	F	159	73	56	__AR	35	21	1	3	20
P30	F	158	61	64	SPG31	15	49	1	0	12
p31	F	149	77	72	SPG31	16	56	2	3	23
p32	M	157	87	59	__AR	30	29	1	2	28
p33	F	150	54	47	SPG7	30	17	1	0	7
p34	M	164	76	32	__AR	14	18	4	4	26
p35	M	170	104	39	__AD	36	3	1	2	12
p36	F	145	43	22	__AD	12	10	3	3	21
p37	M	172	51	17	SPG3A	13	4	1	1	10
p38	M	181	81	28	SPG4	13	15	2	2	12
p39	M	161	78	58	SPG4	43	15	2	3	17
p40	M	177	103	70	SPG4	60	10	2	2	23
p41	M	165	69	28	__AD	20	8	2	3	16
p42	M	186	136	39	SPG3A	20	19	2	2	27
p43	F	161	80	56	SPG4	35	21	2	2	22
p44	M	161	84	62	SPG4	40	22	0	1	5
p45	M	183	78	38	SPG4	30	8	4	4	27
p46	M	175	68	46	SPG7	40	6	1	2	15
p47	M	172	86	43	SPG31	30	13	0	0	2
p48	F	170	65	23	__AR	20	3	0	0	1
p49	M	162	73	51	__AD	46	5	0	2	19
p50	F	159	62	47	SPG4	45	2	0	0	3

AD = autosomal dominant; AR = autosomal recessive; F = female; M = male; SPRS = Spastic Paraplegia Rating Scale; ASH = Ashworth scale of muscle spasticity; __ = molecular diagnosis still not available. The table lists the SPRS scores; higher scores indicate higher disease’s severity.

The control group was fifty healthy subjects (twenty-three women and twenty-seven men, mean age 49.12 ± 11.76 years, height 1.68 ± 0.07 m, weight 70.83 ± 13.22 kg).

All participants provided written informed consent before taking part in the study, which complied with the Helsinki Declaration and had local ethics committee approval (ICOT- Sapienza, Polo Pontino).

### Gait analysis

Kinematic data were recorded at 300 Hz using an optoelectronic motion analysis system (SMART-D System, BTS, Milan, Italy) consisting of eight infrared cameras spaced around the walkway. In accordance with a validated biomechanical model, twenty-two reflective spherical markers (15 mm in diameter) were attached on the anatomical landmarks in accordance with a validated biomechanical model [22], using double-adhesive tape in such a way as to prevent them from falling out of place during the test. In detail, the markers were placed over the cutaneous projections of the spinous processes of the seventh cervical vertebra and sacrum and bilaterally over acromion, anterior superior iliac spine, great trochanter, lateral femoral condyle, fibula head, lateral malleoli and metatarsal head. In addition to markers directly applied to the skin, sticks, or wand, varying in length from 7 to 10 cm, placed at 1/3 of the length of the body segment (femur and leg) were used. Anthropometric data were collected for each subject [23].

Ground reaction forces were acquired by two dynamometric platforms (Kistler 9286B, Winterthur, Switzerland), attached to each other in the longitudinal direction but displaced by 0.2 m in the lateral direction (sampling rate 1200 Hz).

Surface myoelectric signals were recorded at 1000 Hz using a 16-channel wireless system (FreeEMG300 System, BTS, Milan, Italy). After skin preparation, bipolar Ag/AgCl surface electrodes (H124SG Kendall ARBO, Donau, Germany) were placed over the muscle belly in the direction of the muscle fibers according to the European Recommendations for Surface Electromyography [24] and the atlas of muscle innervation zones [25]. Bipolar electrodes, eight in total, were placed on the right side of the body of each subject on the tibialis anterior (TA), gastrocnemius lateralis (LG), gastrocnemius medialis (MG), vastus lateralis (VL), vastus medialis (VM), rectus femoris (RF), biceps femoris (BF), and semitendinosus (ST). Acquisition of kinematic, kinetic, and electromyographic data was integrated and synchronized.

### Experimental Procedure

Patients and controls were asked to walk barefoot at a comfortable, self-selected speed along a walkway approximately 10 m in length while looking forward. Because we were interested in natural locomotion, only general, qualitative instructions were provided. Before the recording session, subjects practiced for a few minutes to familiarize themselves with the procedure. The starting position was adjusted to ensure that the right foot always landed at least on one of the two force platforms embedded in the middle of the pathway. Given that typical walking speeds were slow in these patients, we instructed the control subjects to also walk at a low but comfortable speed. In this way, the general characteristics of gait could be compared between the groups without any potential bias due to speed differences (see below speed matching procedure).

Ten trials per patient were recorded, instead healthy subjects were evaluated for a total of 15 trials (10 trials self selected speed and 5 trials slow walking). To avoid muscle fatigue, blocks of three trials were separated by a one-minute rest period.

### Speed matching procedure

Walking speed was matched between groups as follows: we considered only those control group subjects whose mean walking speed fell within the range identified by patients’ mean walking speed ± SD [26]. Unpaired two-sample t-test was used to investigate differences in walking velocity between patients and controls. In this way, the mean speed values were not statistically different between groups (patients 2.40 ± 1.29 km/h; controls 2.63±0.71 km/h, p = 0.283).

### Data Analysis

Kinematic, kinetic and electromyographic data were normalized to the duration of the gait cycle and interpolated to 201 samples using a polynomial procedure. Gait cycle was defined as the time between two successive foot contacts of the same leg. In this study, heel strike and toe-off events were determined by maximum and minimum of limb angle excursions. Limb angle was calculated as the angle between a vertical axis from the greater trochanter and a vector drawn from the greater trochanter to lateral malleolus projected on the sagittal plane: a 0° limb angle means that the leg was positioned vertically under the body; positive angles denote flexion (i.e. limb positioned in front of the vertical axis) and negative angles denote extension (i.e. limb positioned behind the vertical axis) [27–29]. When subjects stepped on the force platforms, these kinematic criteria were verified by comparison with foot strike and lift-off measured from a threshold crossing event in the vertical force: stance phase was defined as the interval during which the vertical reaction force exceeded 7% of body weight. In general, the difference between the time events measured from kinematics and kinetics was no more than 3% [29] and kinematic criterion proved to be very robust in both healthy subejcts [27] and neurological patients [28]. The raw sEMG signals were band-pass filtered using a zero-lag third-order Butterworth filter (20–450 Hz), rectified, and low-pass filtered with a zero-lag fourth-order Butterworth filter (10 Hz). For each individual, the sEMG signal from each muscle was normalized to its peak value across all trials [29].

Time-distance, kinematic, kinetic and sEMG parameters were evaluated after preprocessing procedures.

#### Time-distance parameters

The following time-distance gait parameters were calculated for each subject: walking speed (km/h), stance duration (% gait cycle), swing duration (% gait cycle), first and second double support duration (% gait cycle), cadence (step/s), step length (% limb length), and step width (% limb length).

#### Kinematic parameters

We computed the anatomical angles for hip, knee, and ankle joints (in the sagittal plane), and trunk and pelvis (frontal, sagittal, and transverse plane). From these variables, we derived the RoM at each joint or segment, defined as the difference between the maximum and minimum value during the gait cycles.

#### Kinetic parameters

Net internal joint moments (Moment_Ankle_, Moment_Knee_, Moment_Hip_) were calculated with an inverse dynamics approach [30] and were normalized to the subject’s body weight. Joint moment curves were used to calculate the angular impulse (AI), i.e., the area under the joint moment curve within a specific time interval [31,32].

Angular impulse quantifies the total contribution of a joint moment to the production of movement and accounts for different gait adaptations (e.g., changes in walking speed) more accurately than peak moment values and it is defined as:

$$\int_{\Delta t}Mdt$$

where M is the flexor-extensor moment of the joint of interest, and Δ*t* is the time interval used to calculate the integral. These angular impulses were hip extensor angular impulse during the first double support subphase (AI_1stDS_Hip_); hip flexor angular impulse during the second double support subphase (AI_2ndDS_Hip_); knee first and second extensor angular impulse (AI_1st_Knee_ and AI_2nd_Knee_ respectively) during the stance phase; ankle dorsiflexor angular impulse during the first double support subphase (AI_1stDS_Ankle_); ankle plantar flexor angular impulse during the mid-stance subphase (AI_MidStance_Ankle_); and ankle plantar flexor angular impulse during the second double support subphase (AI_2ndDS_Ankle_) (S1 Fig). We also evaluated the moment of support (MS) as follow:

MS=MH+MK+MA

calculated as the sum of the moments MH, MK, and MA, which refer to the total curves of Moment_Hip_ Moment_Knee_ and Moment_Ankle_, respectively.

Specifically, we considered the area (MS_Area_) within the gait cycles and the values of the two peaks of the curve (MS_Peak1_ and MS_Peak2_).

sEMG parameters. From the processed EMG signals, we calculated the simultaneous activation by considering the time-varying multi-muscle co-activation function (TMCf) proposed by Ranavolo and colleagues [33]:

$$TMCf\left(d\left(t\right),t\right)=\left(1-\frac{1}{1+e^{-12\left(d\left(t\right)-0.5\right)}}\right).\frac{\;(\sum_{i=1}^{N}EMG_{i}\left(t\right)/\left(100xN\right)){\;}^{2}}{max_{i=1\ldots N}\left[EMG_{i}\left(t\right)\right]}$$

where d(t) is the mean of the differences, N is the number of muscles considered in the analysis, and *EMG*_*i*_ is the sEMG signal of i_th_ muscle. For each subject, data over individual strides were calculated and then averaged across cycles.

As co-activation indices, we considered the area of the TMCf (TMCf_Area_) within the gait cycles. We calculated the TMCf and TMCf_Area_ by considering knee (RF-VL-VM vs BF-ST) and ankle (MG-LG vs TA) antagonistic muscles (TMCf_Knee_, TMCf_Area_Knee_, TMCf_Ankle_, TMCf_Area_Ankle_, respectively).

#### Patients’ subgroups classification

**Fig 1:**
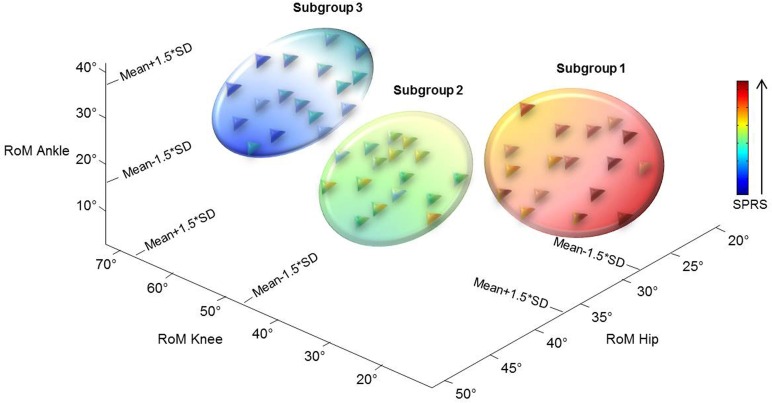
Patients’ subgroups classification according to lower limb joint kinematic behavior. The threshold of mean±1.5*SD of the joint RoMs of the control group is reported. Each patient joint RoM could be either reduced (below the threshold), increased (above the threshold) or close to the values of healthy controls. It is possible to note that the subgroups’ division corresponds also to the severity of the disease scored by SPRS scale (higher values of SPRS correspond to higher disease’s severity). Each triangle represents a patient with different colors according to the SPRS scores. Each circle represents a qualitative characterization of subgroup of patients with different color shades corresponding, in the three spatial dimensions, to the SPRS scores.

In order to classify patients according to their kinematic behavior, we used a z-score with a one tailed z-test for statistical significance [34]. Thus, we chose a z-score of mean±1.5*SD (93% percentile) of the joint RoMs of the control group as the threshold for subgrouping patients with hereditary spastic paraparesis. This z-score threshold is considered as a fairly selective score used in several research fields [35–38]. According to this criterion, each patient joint RoM could be either reduced (below threshold), increased (above threshold), or not significantly different from the values of the healthy controls. Thus, three subgroups of patients were identified. Subgroup one was patients with a statistically significant reduction of RoM at hip, knee, and ankle joints; subgroup two was patients with knee and ankle joint RoMs significantly reduced, but hip joint RoM not significantly different from the control value; and subgroup three was patients with hip joint RoM significantly increased, but ankle and knee joint RoMs not significantly different from the control values (Fig 1).

### Statistical analysis

The Kolmogorov–Smirnov and Shapiro-Wilk tests were used to analyze the normal distribution of the data. Unpaired two-sample t-test or the Mann-Whitney test (two-tailed) were used for between-group differences in the demographic characteristics, time-distance parameters, joint kinematics, joint kinetics and sEMG values. Cohen's d values were also evaluated to estimate the effect size for the comparison between the two means. A multivariate ANOVA was used to compare demographic and clinical parameters (age, gender, disease onset and duration, Ashworth and SPRS scores) between subgroups of patients. One-way ANOVA was used to evaluate the differences in gait variables between the subgroups. Post hoc analyses (with Bonferroni’s corrections) were performed when significant differences were found with the ANOVA. Descriptive statistics included means ± SD, and significance level was set at p<0.05. Lower (LB) and upper (UB) bound of 95% confidence interval are reported for SPRS.

## Results

### Demographic and clinical characteristics

There were no significant differences between the group of patients with hereditary spastic paraparesis and the control group regarding gender, age, weight and height (all p>0.05). Multivariate analysis showed a significant main effect of the subgroup (F_(14,84)_ = 4.468, p<0.001). Specifically, we observed significantly lower values of hip-spasticity Ashworth-score in both subgroups two (mean ± SD: 1.25 ± 0.86) and three (0.82 ± 0.88) as compared with subgroup one (2.23 ± 1.15) (p = 0.017 and p<0.001, respectively); significantly lower values of knee-spasticity Ashworth-score in subgroup three (0.88 ± 0.93) than both subgroup one (2.47 ± 1.12) and subgroup two (2.06 ± 0.68) (p<0.001 and p = 0.002, respectively); significantly lower values of total SPRS score in both subgroup two (16.07 ± 6.48, LB: 12.62, UB: 19.52) and subgroup three (5.64 ± 5.36, LB: 2.89, UB: 8.40) than subgroup one (21.33 ± 7.17, LB: 17.65, UB: 25.02), as well as in subgroup three than subgroup two (p = 0.049, p<0.001, p<0.001, respectively). No significant differences between subgroups were found for any other variable.

### Time-distance parameters

**Fig 2:**
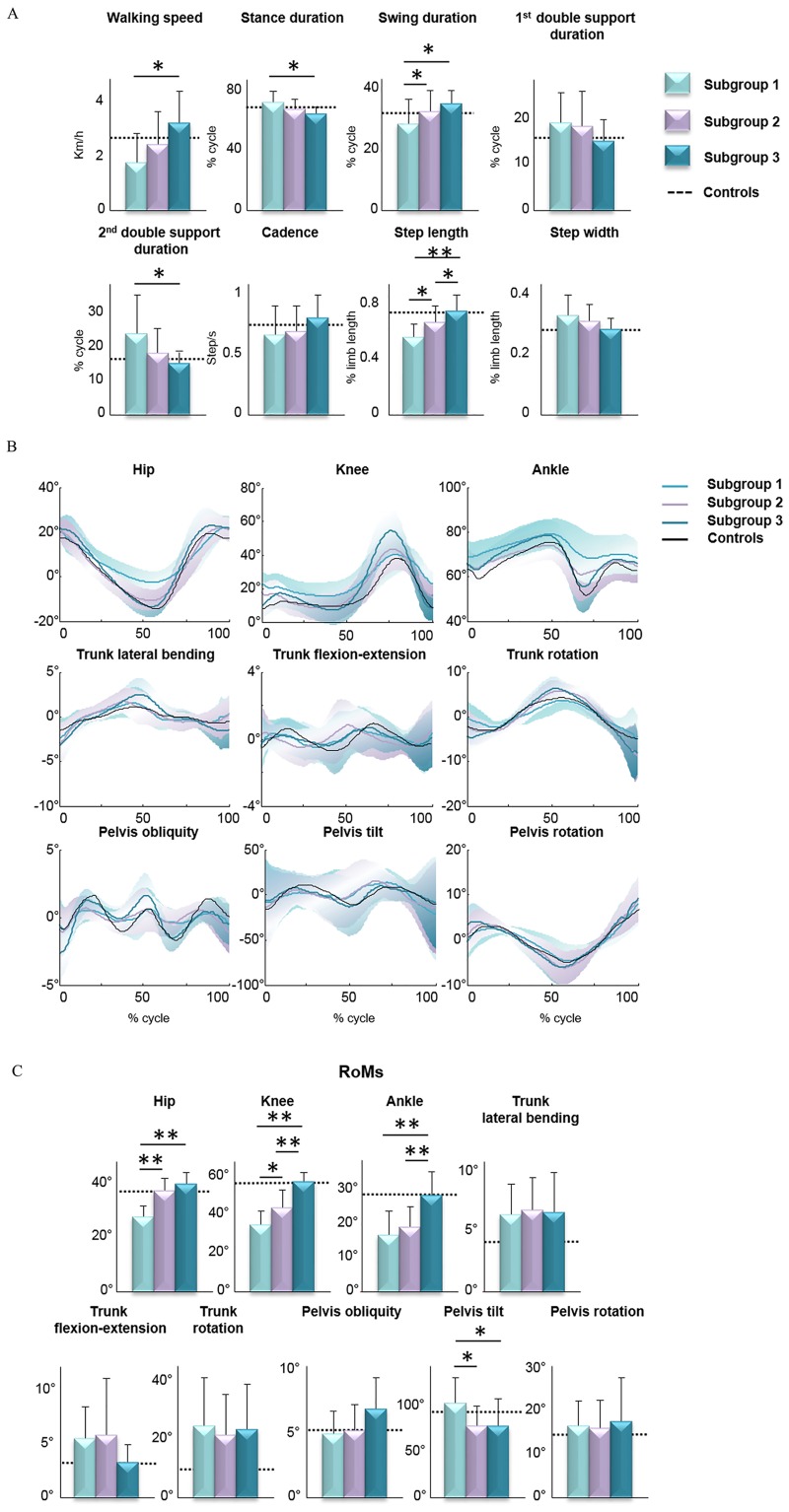
Time-distance and joint and trunk kinematic parameters in HSP subgroups. (A) Mean values (±SD) of time distance parameters. (B) (B) Mean (with SDs in light colors) kinematic plot of joint angular displacements during the gait cycle. (C) Mean values (±SD) of range of angular motion (RoM). Mean values of healthy controls for both time-distance and kinematic parameters, are reported in each bar graph (dotted line) and plot (black line). Asterisks indicate significant differences among the three subgroups at post hoc analysis (* p<0.05, ** p<0.001).

When comparing the whole sample of patients with the healthy participants, no significant differences were found in any time-distance parameters, except for step width and step length, whose values were significantly increased and reduced, respectively, in patients compared with controls (Table 2). A significant effect of patients’ subgroup was found, using one-way ANOVA, on most of the time-distance parameters. These were the main effect of walking speed (F_(2,47)_ = 6.703, p = 0.003); stance duration (F_(2,47)_ = 4.923, p = 0.011); swing duration (F_(2,47)_ = 4.900, p = 0.012); second double support duration (F_(2,47)_ = 4.551, p = 0.016); and step length (F_(2,47)_ = 11.173, p<0.001). Post-hoc analysis revealed significantly higher values of walking speed in subgroup three than in subgroup one, lower stance duration in subgroup three than in subgroup one, higher swing duration in both subgroups two and three than in subgroup one, lower second double support duration in subgroup three than in subgroup one and higher step length in subgroup three than in both subgroups one and two and in subgroup two than in subgroup one (Fig 2A).

**Table 2:** Time-distance, kinematic, kinetic and sEMG mean±SD data in patients and controls.

	Patients	Controls		
	Mean±SD	Mean±SD	P values	Cohen’s d
***Time-distance parameters***				
Walking Speed [km/h]	2.40±1.28	2.63±0.70	0.283	0.223
Stance duration [% cycle]	68.33±7.16	67.21±2.54	0.622	0.208
Swing duration [% cycle]	31.76±7.19	32.92±3.11	0.657	0.209
1^st^ double support [% cycle]	18.18±6.99	16.85±2.79	0.554	0.250
2^nd^ double support [% cycle]	19.07±9.14	16.91±3.34	0.434	0.314
Cadence [steps/s]	0.71±0.23	0.70±0.12	0.120	0.055
Step length [% limb length]	**0.66±0.14**	**0.72±0.09**	**0.023**	**0.510**
Step width [% limb length]	**0.31±0.06**	**0.28±0.05**	**0.004**	**0.543**
***Kinematic parameters (Rangeof angular motion)***				
Hip [°]	35.10±6.78	34.96±4.63	0.905	0.024
Knee [°]	**45.45±11.90**	**56.35±5.95**	**<0.001**	**1.159**
Ankle [°]	**21.36±8.57**	**27.79±6.79**	**<0.001**	**0.832**
Trunk lateral bending [°]	**6.67±2.93**	**3.72±1.70**	**<0.001**	**1.232**
Trunk flexion-extension [°]	**4.39±2.61**	**2.92±0.76**	**0.004**	**0.765**
Trunk rotation [°]	**25.29±17.45**	**13.16±14.42**	**<0.001**	**0.758**
Pelvis obliquity [°]	6.09±2.28	5.16±1.56	0.298	0.476
Pelvis tilt [°]	89.14±31.50	90.29±28.44	0.931	0.038
Pelvis rotation [°]	**16.89±7.86**	**13.77±8.32**	**0.007**	**0.385**
***Kinetic parameters***				
AI_1stDS_Hip_	8.24±3.52	10.36±2.54	0.568	0.691
AI_2ndDS_Hip_	2.93±2.91	2.24±1.71	0.348	0.289
AI_1st_Knee_	**3.02±4.02**	**2.53±4.08**	**0.016**	**0.121**
AI_2nd_Knee_	2.14±3.92	1.05±1.91	0.642	0.353
AI_1stDS_Ankle_	0.91±3.31	0.98±2.09	0.917	0.025
AI_MidStance_Ankle_	27.55±8.07	28.22±5.19	0.063	0.099
AI_2ndDS_Ankle_	8.63±5.22	7.03±3.07	0.716	0.374
MS_Area_	45.98±17.43	41.64±14.48	0.332	0.271
MS_Peak1_ [N*m/Kg]	1.09±0.34	1.02±0.41	0.324	0.186
MS_Peak2_ [N*m/Kg]	0.92±0.39	0.86±0.29	0.526	0.175
***sEMG parameters***				
TMCf_Area_Ankle_	**21.20±6.39**	**13.20±3.70**	**<0.001**	**1.532**
TMCf_Area_Knee_	19.12±7.62	16.62±4.41	0.380	0.402

Bold type indicates significant differences between patients and controls. Cohen’s d values indicate the effect size for the comparison between the two means ("small" if d = 0.2, "medium" if d = 0.5, "large" if d = 0.8).

### Kinematic parameters

Significant lower values in knee and ankle RoMs and significant higher values in trunk lateral bending, flexion-extension, and rotation RoMs and pelvis rotation RoM were found in patients than in controls (Table 2). A significant main effect of the subgroup was found, using one-way ANOVA, on hip (F_(2,47)_ = 33.747, p<0.001), knee (F_(2,47)_ = 38.555, p<0.001), ankle (F_(2,47)_ = 14.043, p<0.001), and pelvis tilt (F_(2,47)_ = 4.328, p = 0.019) RoMs. Post-hoc analysis revealed significant higher values in hip RoM in both subgroups two and three than in subgroup one, higher values of knee RoM in both subgroups two and three than in subgroup one and in subgroup three than in subgroup two, higher values of ankle RoM in subgroup three than in both subgroups one and two, and lower values of pelvis tilt RoM in both subgroups two and three than in subgroup one (Fig 2B and 2C).

### Kinetic parameters

**Fig 3:**
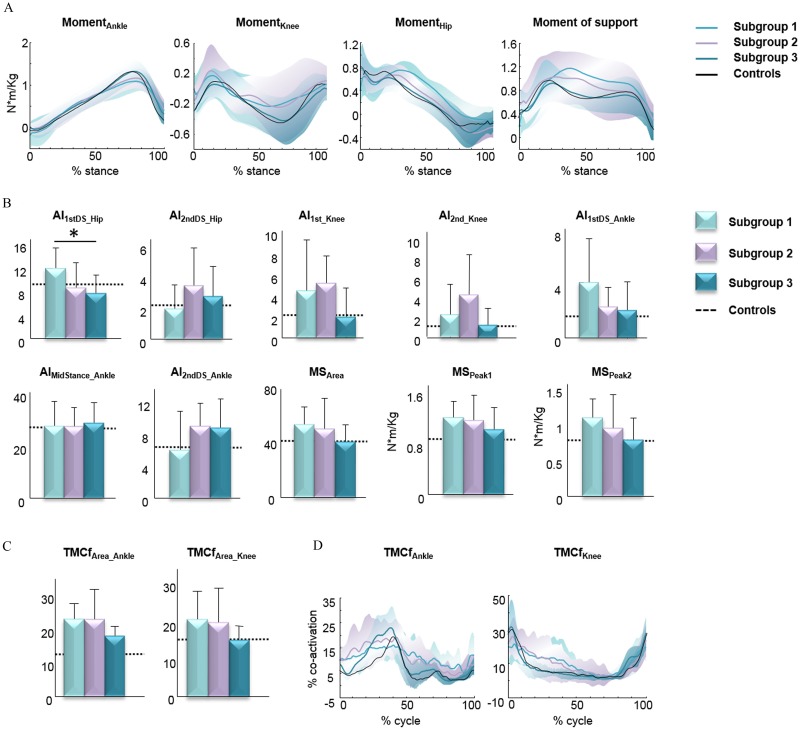
Joint kinetic and muscles parameters in HSP subgroups. (A) Mean (with SDs in light colors) kinetic plot of joint moments (hip, knee and ankle) and support moment. The patterns are normalized to body weight and plotted vs. normalized stance. (B) Mean values (±SD) of kinetic parameters. (C) Mean TMCf_Area_ (±SD) calculated for ankle and knee muscles (TMCf_Area_Ankle_ and TMCf_Area_Knee_). (D) Mean (with SDs in light colors) plot of co-activation of ankle and knee joint antagonist muscles during the gait cycle. Mean values of healthy controls for kinetic and muscles parameters are reported in each bar graph (dotted line) and plot (black line). Asterisks indicate significant differences among the three subgroups at post hoc analysis (* p<0.05).

Significant differences were found only for AI_1st_Knee_whose value was higher in patients than controls (Table 2). A significant effect of the subgroup was found, using one-way ANOVA, on AI_1stDS_Hip_ (main effect, F_(2,47)_ = 3.517, p = 0.043). Post-hoc analysis showed lower values of this parameter in subgroup three than subgroup one (Fig 3A and 3B).

### sEMG parameters

Significant higher values in TMCf_Area_Ankle_ were found in patients than controls (Table 2). No significant effect of the subgroup was found, using one-way ANOVA, on sEMG parameters (Fig 3C and 3D).

## Discussion

We investigated the gait patterns in patients with hereditary spastic paraparesis by performing a comprehensive analysis of all time-distance, kinematic, kinetic, and sEMG parameters. In particular, our study was aimed at identifying specific subgroups of patients according to their kinematic behavior. Our assumption herein was that the decrease in the joint RoMs reflected the presence and extent of spasticity, and thus the primary deficit characterizing the gait of patients with hereditary spastic paraparesis. Few studies have previously investigated the gait in adults or children with hereditary spastic paraparesis [18–20]. In line with these previous studies, we found an abnormal gait pattern characterized by reduced step length, increased step width, and reduced RoM at the knee joint in the whole sample of patients as compared with the control group. Furthermore, we found increased trunk RoM in all three spatial planes, increased pelvic tilt, increased hip joint torques (AI_1st_Knee_), reduced ankle joint RoM, and increased co-activation of muscles acting at the ankle joint. In addition to these general biomechanical characteristics of gait, one would expect some differential characteristics in distinct subgroups of patients according to clinical involvement of the pyramidal tract, given that patients with hereditary spastic paraparesis exhibit different degrees of severity both within and between families [1]. Thus, some specific biomechanical features may not emerge because they are hidden within their global walking strategy. Compared to previous studies, we enrolled a greater sample of patients with HSP (fifty in our study compared with twenty-two [18], nine [19] and twenty [20]) and performed an overall analysis of time-distance, kinematic (upper and lower body), kinetic and sEMG parameters. This allowed us to identify subgroups of patients and to define a global picture of walking strategies adopted by them. When subgrouping patients according to the hip, knee and ankle joint kinematic behavior, three clear gait patterns emerged. The gait pattern of subgroup one was characterized by reduced RoMs at hip, knee and ankle joints. Patients of this subgroup were the most severely affected (highest SPRS score) (Fig 1), and walked at the slowest speed. The gait pattern was characterized by the highest stance and second double support durations and the shortest swing duration and step length (Fig 2A). Such gait pattern reflects on one hand the reduced gait speed; on the other hand, the attempt to increase the most stable configuration duration (bipedal support), aimed at maintaining the dynamic balance. Furthermore, in these patients, we observed increased values of RoM for pelvis tilt and hip extensor angular impulse during the first double support subphase (Figs 2C and 3A and 3B). The former result might be due to spasticity and contracture of hip muscles as reported in neurological disorders with lower limb spasticity [20], [39–41]. The last result indicates that HSP patients, although they have reduced hip RoMs, need to greatly involve the hip joint for weight acceptance increasing the internal torques. Interestingly, this finding further reinforces the notion that spasticity predominates on muscle weakness in the most severely involved patients [42,43]. The gait pattern of subgroup three was characterized by increased hip joint RoM and knee and ankle joint RoMs close to control values. These patients were the most mildly affected (lowest SPRS score) (Fig 1) and showed the highest walking speed. Their gait pattern included the highest swing duration and step length, the shortest stance and second double support duration values (Fig 2A) and the lowest pelvis tilt RoM and hip extensor angular impulse (during the first double support sub phase) than the other subgroups (Figs 2C and 3A and 3B). From Fig 2, it is possible to note that patients of this subgroup showed a gait pattern which was close to that of healthy controls in terms of time-distance parameters. It is also important to note that, with respect to healthy subjects, this subgroup of patients showed increased trunk RoM in all three spatial planes. Considering the very low SPRS score in this subgroup, this result suggests that the compensatory mechanisms represented by the increased trunk movements and hip RoM are the most important biomechanical features characterizing the gait disorders from the early phase of the disease. Patients of subgroup two had characteristics between those of subgroups one and three, in terms of disease severity (Fig 1) and gait speed, and showed hip joint RoM close to controls but decreased knee and ankle joint RoMs. In particular, their gait pattern was characterized by intermediate values with respect to the other two subgroups in terms of step length, swing duration and pelvis tilt RoM (Fig 2).

As regards the sEMG, we observed significantly increased co-activation of antagonist muscles acting at the ankle for the whole group of HSP patients compared with healthy controls (Table 2). When analyzing patients’ subgroups, a trend, although not statistically significant, for higher co-activation values of knee and ankle antagonist muscles was also observed (Fig 3C and 3D). Such a finding reflects the inability of the CNS to selectively activate lower limb joint muscles and may be explained by inefficient mechanisms of reciprocal inhibition [44] and the supraspinal and spinal plastic neuronal changes associated with the development of spasticity [45,46]. In general the different patterns of gait disturbance seem to correlate fairly well with the different degree of disease’s severity among patients, as measured by the SRPS score, with mild (SPRS < ten), moderate (SPRS < twenty) and more severe presentation (SPRS > twenty). In addition, since we chose to study only patients with pure pyramidal signs, regardless of the genetic form (namely SPG3A, 4, 5, 7 and 31), we can safely assume that the identified three different gait patterns based on lower limb kinematic behavior, also reflect the different degree of pyramidal tract involvement in individual patients. We think that identifying specific gait patterns [41] in patients with hereditary spastic paraparesis may be useful in: i) improving our understanding on gait disorder in hereditary spastic paraparesis by sorting out the most meaningful gait features from the complexity of locomotion; ii) recognizing specific abnormalities and their impact on clinical decision-making; and iii) individualizing rehabilitative treatment and better evaluating its effects over the time.

## Supporting Information

S1 Data Main data underlying the findings described in the manuscript. (XLSX)

S1 Fig Method of angular impulse evaluation on hip, knee, and ankle joint moments curves. (TIF)
