# Supplementary figures and images for: Gait Patterns in Patients with Hereditary Spastic Paraparesis
Source: PLoS One. 2016 Oct 12;11(10):e0164623. doi: 10.1371/journal.pone.0164623 (PMC5061421; doi:10.1371/journal.pone.0164623)

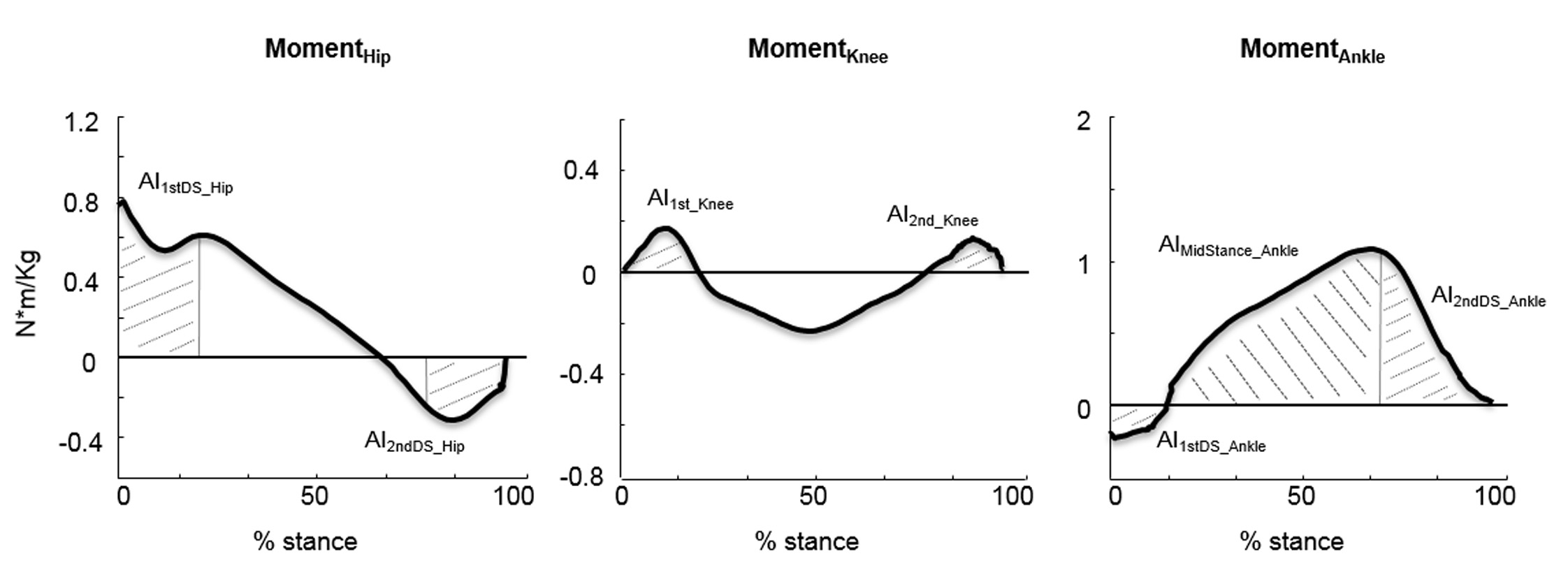

Supplement: S1 Fig — (TIF) [file pone.0164623.s002.tif]
